# A resilient health system? Well-child visits before and after COVID-19 in Chile

**DOI:** 10.3389/fped.2025.1497358

**Published:** 2025-03-14

**Authors:** Carolina Acevedo

**Affiliations:** Research, Innovation and Creation Center (CIIC-UCT), Temuco Catholic University, Temuco, Chile

**Keywords:** health system resilience, COVID-19, well-child visits, recovery rate, primary health care

## Abstract

**Introduction:**

Childhood is a critical period where access to medical controls and health policies can severely affect health equity.

**Objectives:**

Analyzed to subnational Chile health system's resilience in the face of the COVID-19 pandemic regarding child health. The focus of the research was to assess the performance of Chile Crece Contigo's social policy, specifically the Well-Child Visit Program, which provides health checks to one million children annually through the primary health network.

**Methods:**

Using a subnational post-pandemic recovery rate, a regression analysis with municipal controls was performed to observe recovery facilitators. A percentage based on the total child population was obtained for the commune of each country and year of study (2015–2022). Data was collected from the Monthly Statistical Reports (REM) of the Ministry of Health, the Socioeconomic Characterization Survey (CASEN), and the National Institute of Statistics of Chile (INE).

**Results:**

Recovery rate at the national level was −3.2%. However, when examining the subnational reality, it was found that 66% of Chilean municipalities are unable to reach pre-pandemic figures and are also territorially concentrated in certain regions of the country. The analysis revealed that municipalities with the worst recovery rate results had negative health expenditure per population.

**Conclusions:**

Examining this phenomenon through a subnational lens invites contemplation on the importance of contextualizing it within the theoretical framework of health system resilience. The child health system's capacity must build knowledge based on developing public policies, governance, financing, and coordination in the territories to overcome the crisis.

## Introduction

The COVID-19 pandemic was classified as a biomedical, social, and economic catastrophe (1). The inequalities between countries and the different groups that make up society have resulted in significant disparities in confronting and enduring this crisis. Consequently, the pandemic has imposed exceptional challenges on the capabilities of global healthcare systems, with certain countries demonstrating higher effectiveness levels than others (2–4).

As we approach the first year since the implementation of vaccination protocols and the gradual easing of restrictions, it is essential to recognize the need to pay greater attention to other health concerns and vulnerable demographic groups, such as neonates and childhood (0–9 years), who are of significant concern in terms of medical controls, vaccination processes and screening for diseases at an early age (5–8). In 2022, the United Nations (UN) expressed the need for health systems to focus on caring for the most vulnerable groups in society, including women, children and neonates, in order to avoid jeopardizing progress in health coverage (9). In response to this important call, this article delves into the relevance of assessing the resilience of Chile's health system to safeguard children's health.

The concept of resilience was frequently used in cases of natural disasters, but Data was collected from the Monthly Statistical is relatively new when applied to health systems. A crucial element for implementing this idea within health systems is its ability to enhance healthcare institutions and professionals' preparedness, recovery, and absorption of crises (10). It is important to emphasize that resilience involves restoring the previous state and advancing toward a better approach to confronting future crises. Resilience, therefore, encompasses growth and the ability to bounce back from adversity (11). Thus, the child health system's capacity must build knowledge based on developing public policies, governance, financing, and coordination in the territories to overcome the crisis.

The case of Chile presents a unique perspective, particularly concerning the investment in children through the *Chile Crece Contigo* ChCC (Chile Grows with You) subsystem over the past 15 years. This initiative, abbreviated as ChCC, focuses on promoting preventive medicine by offering various health programs in primary health care centers. Among these programs is the Well-Child Visit Program, which was studied here. This checkup program provides and promotes medical checkups, including biopsychosocial risk assessments and physical examinations, to children aged 0–9 years. Therefore, assessing the system's resilience in access to child health services in primary healthcare centers is crucial to understanding how the resources installed in the territories have helped to withstand the pandemic and its effects on child healthcare access.

Therefore, assessing system resilience in access to child health services in primary health care centers is crucial to understand how resources deployed in territories have helped withstand the pandemic and its effects on access to child health care. Since preventive health is coordinated and funded at this level, considering a subnational perspective is essential when assessing the resilience of health systems in countries where administrative processes, financing, capacity, and medical personnel are distributed across smaller territories. This approach allowed for a more comprehensive understanding of the resilience-building process.

## Methodology

To assess the extent to which the child health care system has returned to pre-pandemic levels, the recovery rate was calculated based on non-attendance at the Well-Child Visit Program. This program falls within the *Chile Crece Contigo* ChCC (Chile Grows with You) subsystem and provides primary healthcare for children from birth to 9 years old. The data were collected from the Monthly Statistical Reports (REM) of the Ministry of Health, the Socioeconomic Characterization Survey (CASEN) and the National Institute of Statistics of Chile (INE). The gross value of non-attendance was determined by dividing it by the population up to 9 years old, according to data from the National Institute of Statistics (INE). Then, a percentage was calculated based on the total child population for the commune and year of study of each country (2015–2022). From a methodological point of view, it is important to consider that a commune represents the smallest administrative subdivision in Chile. Based on the information provided to the REM, CASEN and INE, the recovery rate of the child health system for the next year 2022 was obtained.^1^

Two variables define this rate:

*P*(*i*) refers to the percentage of non-attendance of Well-Child Visit Program, considering the communes with data on non-attendance to these check-ups between 2015 and 2019.^2^

Q (2022) refers to the percentage of non-attendance of Well-Child Visit Program for 2022, but only for communes with no missed cases during that year.

(1) $$\mathrm{Recovery}\;\mathrm{Rate}\;=\frac{1}{5}\;\;\overset{2019}{\underset{\;i=2015}{\sum }}\;P(i)\;\;-\;\;Q(2022)$$

Taking previous studies (12, 13), the recovery rate will be classified according to the following criteria: (1) Positive (2%–15%): The controls have improved their rate of attendance at child check-ups compared to the period before the pandemic; (2) Null (−1% to 1%): There are no significant changes in attendance at child check-ups; (3) Negative (−2% to −40%): Indicates that the commune has not recovered the levels of attendance at child check-ups prior to the pandemic.

Analyzing data at the commune level provides insight into the conditions surrounding preventive health services. This approach also facilitates the examination of underlying factors that impact the population and are closely tied to social determinants of health and governance processes at the local government level. Such factors could help explain why certain local governments have experienced varying recovery levels. While some studies have developed similar index designs (14), their focus has been on assessing previous resilience capabilities rather than on recovery aspects of the health system (15).

Explanatory variables were analyzed based on the health resilience framework (2, 16) which includes various indicators related to governance, financing, health worker capacity, and healthcare services. Specifically, the following variables were used considering the limitation of data at the subnational commune level: Rurality; rate of women; poverty in the commune; health expenditure in the commune; health professionals in the commune; medical personnel in the commune and health training. Data sources include the National Municipal Information System (SINIM), developed by the Undersecretariat of Regional and Administrative Development, which offers a wide range of indicators, the CASEN survey, and the INE, for the years 2015–2022.

To determine the explanatory variables, a consistent formula was used to calculate the difference rate (Equation 1). New variables were generated by subtracting the average value between 2015 and 2019 from the value in 2022. This approach allowed us to assess the impact of these indicators on the health system's recovery rate, specifically regarding Well-Child Visit Program. Table 1 shows a breakdown of the descriptive statistics associated with the variables we considered.

**Table 1:** Descriptive statistics of explanatory variables.

Variables	Obs.	Mean	Std. Dev
Difference rate % of poverty in the commune (CASEN)^a^.	345	12.62543	6.16922
Difference rate % of rurality in the commune (CASEN)^a^.	345	.931947	2.475002
Difference rate % women in the commune (SINIM)^b^.	345	−.0197084	.1936317
Difference rate % of expenditure on training of human resources in health (Population according to MINSAL^c^ Annual Decree).	319	.0427247	.3599339
Difference rate of annual health expenditure per inhabitant in the commune (SINIM)^b^.	319	852.021	44,872.84
Difference rate of physicians in the municipality per 10,000 inhabitants (SINIM)^b^.	298	−2.357077	17.01384
Difference rate % of municipal professionalization in the commune.	335	−6.767699	8.078075

Source: Own elaboration with data from REM, INE and CASEN.

^a^ Socioeconomic characterization survey (CASEN).

^b^ National system of municipal information (SINIM).

^c^ Ministry of health (MINSAL).

## Results

Data from the REM reports for the years preceding the pandemic (2015–2019) indicated that the national average of non-attendance of Well-Child Visit Program was close to 10%. This percentage has increased significantly in 2020 and 2021, with a peak average of 18.4%. Such a trend was not observed in previous years, which is alarming given the initial success of the ChCC subsystem. Furthermore, the recovery rate in 2022 at the country level was −3.2% compared to the 2019 results, which is even higher than the average rate the program had been showing in previous years.

**Figure 1:**
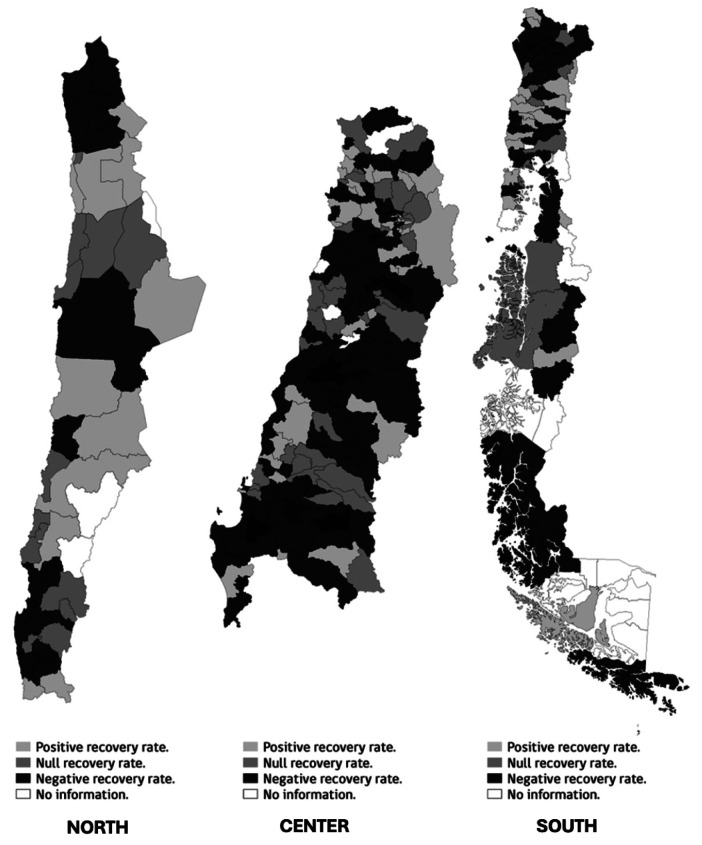
Distribution of the Well-Child Visit Program recovery rate by communes in Chile. Source: Own elaboration with data from REM and INE. REM, monthly statistical reports; INE, National Institute of Statistics of Chile.

Of the 325 communes analyzed, 216 did not achieve a positive recovery rate, indicating that 66% of communes in Chile experienced an increase in the percentage of children who stopped attending the Well-Child Visit Program compared to pre-pandemic levels. Figure 1 shows the geographical distribution of the communes divided into north, center and south, and is divided into three categories according to their recovery rate: positive (2%–15%), zero (−1% to 1%) and negative (−2% to −40%). The distribution of data in the center of the country and the first region of the southern zone, the Araucanía region, showed predominantly negative recovery rates, while the northern regions show disparate results between the different communes.

**Figure 2:**
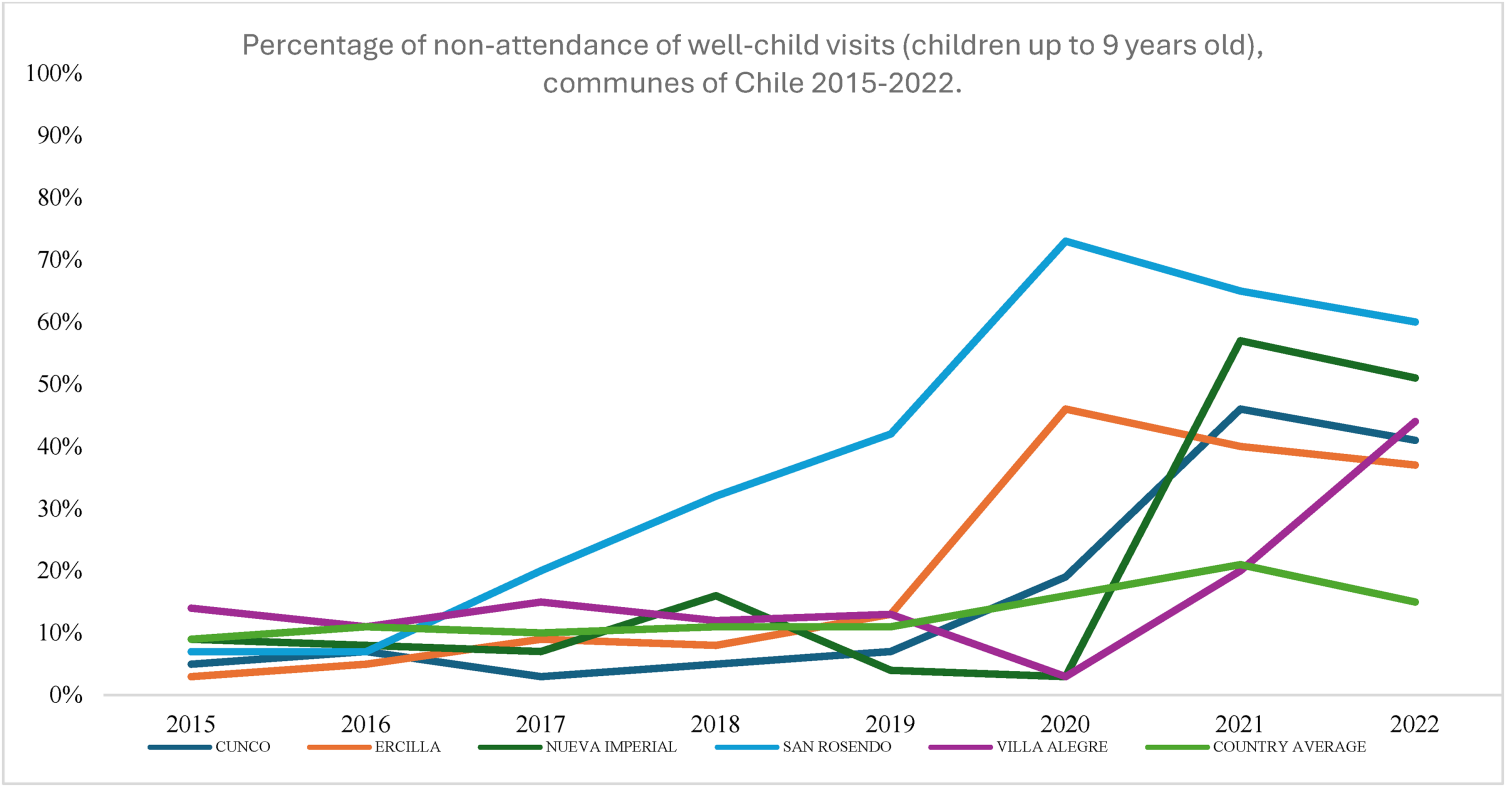
Percentage of non-attendance of Well-Child Visit Program (children up to 9 years old), communes of Chile 2015–2019. Source: Own elaboration with data from REM and INE. REM, monthly statistical reports; INE, National Institute of Statistics of Chile.

Figure 2 shows the percentage of non-attendance of Well-Child Visit Program in the five municipalities with the lowest recovery rates. Unfortunately, this percentage has increased from 2019 to 2020 due to the pandemic, and even by 2022, the figures have yet to return to pre-pandemic levels. Notably, three of these five communes are in La Araucanía, one of the country's poorest regions.

To complement the above analysis, a regression analysis with common controls was performed to uncover potential factors that may contribute to the recovery rate. As mentioned above, a pre- and post-pandemic difference rate was calculated for each independent variable to account for any fluctuations in variables such as rurality, poverty, health expenditure, and health personnel, all of which the World Health Organization currently considers in terms of health system resilience (16). Table 2 displays the model's findings. It is worth noting that the lack of subnational data is a significant limitation in studying Chile's health system. Of the 346 communes' areas, only 280 have complete information recorded in institutional systems that allowed for a response analysis such as the one presented here. As a result, the covariates that demonstrate the greatest strength in this regard and align with the theoretical guidelines presently evaluated in terms of installed capacities within health systems.

**Table 2:** Predictors of recovery rate access to child health services care.

Variables	Model 1
Difference rate of rurality in the commune	−0.505
	(−1,79)
Difference rate of women in the commune	−2.345
	(−0,82)
Difference rate of poverty in the commune	−0,161
	(−1,51)
Difference rate of health expenditure in the commune	−0,0000266**
	(−3.11)
Difference rate of health professionals in the commune	0.0206
	−0,31
Difference rate of medical personnel in the commune	−1.021
	(−0,85)
Difference rate of health training	−0.0102
	(−0,84)
Constant_	−2.012
	(−1,39)
*N*	280

*T* statistics in parentheses. Source: Own elaboration with data from REM^a^, SINIM^b^ and CASEN^c^. REM, monthly statistical reports; SINIM, National System of Municipal Information; CASEN, socioeconomic characterization survey.

* *p* < 0.05; **p < 0.01; ***p < 0.001.

Table 2 shows the regression model where different predictors that could influence the rate of recovery of access to children's health in the different communes of Chile are evaluated. The “Model 1” column offers the magnitudes of each independent variable on the recovery rate. The only variable that showed statistical significance is the health expenditure rate in the commune with a coefficient of −0.0000266 and a t statistic of −3.11. This suggests that as the difference in health expenditure between the pre- and post-pandemic years increases, the dependent variable (the post-pandemic recovery) decreases. The result of the other covariates, although it has a direction consistent with the theory, there is no statistical significance that allows us to conclude any causal relationship.

## Discussion

The resilience of health systems became an urgent concern in the post-pandemic era. Addressing this issue is crucial because it allows us to learn from the crisis at the institutional level and because it has far-reaching implications for societies once the fear of contagion subsides. This analysis addressed the resilience of the child health system in Chile, considering how the COVID-19 pandemic affected access to child health services checks. The “Chile Crece Contigo” program, specifically the well-child check-up, has demonstrated substantial progress in child health in recent decades; however, the health crisis exposed weaknesses in the system's resilience, especially at the subnational level.

Over the past 60–70 years, public health performance for children in Chile has achieved remarkable success (17). Starting from the first Child Protection Congress in 1920 and extending to the expanded immunization program in 1970, public health policies in Chile have continuously strived to enhance children's well-being. As a result, the country has established a prominent position in Latin America for its maternal and child public health, particularly in maternal mortality indicators and child vaccination processes. Nevertheless, it was not until the implementation of the “*Chile Crece Contigo* (ChCC)” subsystem during the first government of Michell Bachelet that primary healthcare figures and controls were solidified.

The ChCC subsystem was designed to safeguard the physical, psychological, social, and biological well-being of children and their families from pregnancy to age 9. Over the years, the program has undergone several reforms and enhancements, making it one of the most extensive public policies in the region. It can be integrated into the primary healthcare system and provides comprehensive care from prenatal checkups to pre-adolescence. The program supports nearly 200,000 expecting mothers and almost one million children annually, focusing on serving the most vulnerable families (18).

The ChCC program prioritizes the health and well-being of families by implementing tasks and interventions that detect potential health issues early on. Primary health centers (PHCs) play a crucial role in diagnosing these pathologies quickly and efficiently. One of the central interventions within the program is the Well-Child Visit Program (*Control de Niño Sano* in Spanish). The Ministry of Health website states that “this service aims to accompany children's comprehensive development in physical, emotional, social, cognitive, and language areas” (19). This service includes a range of assessments, such as a general and biopsychosocial risk background check, cephalocaudal physical examination, dental checkup, visual, hearing, and respiratory screening, and a review of the vaccination plan. The ChCC also coordinates a health checkup calendar with primary health services to ensure that each commune reaches its health goals based on its population enrolled in municipal PHCs.

The attainment of these goals is influenced by the institutional and territorial environment where the ChCC initiative is implemented. The decentralized primary PHC level is funded and managed by local governments (municipalities) with varying institutional and governance abilities in different regions, leading to heterogeneous performance. This poses a challenging institutional scenario, especially considering the program's reliance on integrating existing resources in each territory. Every family healthcare center (CESFAM, for its acronym in Spanish) and primary healthcare center offers services based on their available resources, whether centralized or decentralized.

Several studies examined the effects of the ChCC program on children's health and have noted significant advancements (18, 20). Research has shown that the program has increased the average birth weight of babies by approximately 10 g, with even more positive outcomes in families who are more vulnerable (16, 21). However, it is worth noting that the program's impact is most significant in families with a lower vulnerability score [as assessed by the *Ficha de Protección Social* (Social Protection Form)] (18, 22). Additionally, other studies have highlighted the ChCC program's success in reversing negative trends following the 2010 earthquake in Chile, thanks to its multisectoral approach and strategic interventions that enabled continuous care (23).

The results of the study highlight a successful public health program in Chile that has gained international recognition for its praiseworthy performance. However, the pandemic has shifted the focus from the Ministry of Health to tertiary care centers such as hospitals and emergency centers. This has resulted in a lack of coordination within the network and a failure to adequately protect children's health.

The findings indicated a concerning lack of resilience in the health system, with only 34% of municipalities successfully recovering from the pandemic crisis. The analysis reveals that the municipalities with the poorest outcomes can largely attribute their struggles to disparities in health expenditure per population. This aligns with the ongoing development of primary health in Chile, which encompasses numerous programs under the ChCC subsystem, mainly focused on children's health monitoring. Consequently, the observed variations in funding systems are not a novel phenomenon but a daily reality that each municipality must confront. To effectively implement a national policy, municipalities must acknowledge and address their respective territories’ diverse capacities and realities, recognizing the inherent heterogeneity.

The difference in spending is notable since the financial aspect, or the spending allocated to the health sector, is a fundamental factor in the resilience of health systems. The fact that the system's care and capabilities are focused on reversing the results of the pandemic in the general population, left primary health centers without financial provision, which, despite having fewer resources, were unable to regain pre-pandemic care figures.

Currently, each municipality in Chile receives a monthly state contribution from the Ministry of Health via the relevant Health Services. This contribution amounts to around US$ 8 per person and is based on factors such as the beneficiary population, socioeconomic status, rurality indexes, and challenges in delivering healthcare per commune. Additionally, the State provides an additional 60% of the municipal budget to support primary healthcare through initiatives like *Chile Crece Contigo* (Chile grows with you), *Equidad en Salud Rural* (Equity in Rural Health Program), *Sistema de Urgencia Rural* (Rural Emergency System), and *Rehabilitación Integral de Base Comunitaria* (Comprehensive Community-Based Rehabilitation), among others.

Examining the health system's resilience through a subnational perspective sheds light on the vulnerabilities present in the health system of Chile. The diverse nature of communes regarding their socioeconomic and territorial characteristics underscores the importance of establishing a coherent framework for distributing healthcare funds, particularly in times of crisis. Furthermore, effective national social programs like the ChCC must secure adequate funding to mitigate risks and consider the unique commune dynamics to ensure efficient collection and distribution of resources across different contexts. The World Health Organization emphasizes the significance of financial policies that promote resilience by addressing the equitable mobilization of funds, often requiring a progressive approach to funding allocation.

Resilience in health systems became an urgent concern in the post-pandemic era. It is crucial to address this issue because it allows us to learn from the crisis at an institutional level and because it has far-reaching implications for societies once the fear of contagion subsides. The impact on governance and public policy must be considered, especially considering the setbacks experienced by successful programs like the ChCC program due to the pandemic. As a result, it is observed that the “Chile Crece Contigo” program is diminished by the structural conditions of subnational politics. In this sense, resilience becomes a necessity. In the current context of Chile, there is a demand for flexibility and adaptation to the circumstances of the different places that make up the health system. Therefore, place-based resources become the roadmap for how the system should face the crisis.

Examining this phenomenon through a subnational lens invited contemplation on the importance of contextualizing it within the theoretical framework of health system resilience. Understanding the unique intricacies of various health systems across countries is pivotal. It goes beyond individual actors and encompasses the community's overall performance and the historical changes that often account for their response to crises.

Finally, it was important to mention that the study had limitations related to the availability of subnational data that allow the addition of other covariates to the design that may allow a better understanding of what is happening with the recovery rate. Likewise, measuring health resilience is comprehensive given the multidimensional nature of the concept, so the operationalization could take different forms depending on the preferences to observe the phenomenon. Advancing in qualitative studies that allow us to understand what has happened with primary health care and the healthy child program is an interesting way to advance in new variables that allow us to understand how to recover health systems in the face of health catastrophes such as the COVID-19 pandemic.

## Data Availability

The original contributions presented in the study are included in the article/Supplementary Material, further inquiries can be directed to the corresponding author.

## Footnotes

^1^It is indicated that of the 346 communes existing in Chile for the years of study, only 325 present data to calculate the recovery rate.

^2^It is important to consider that the first reported case of coronavirus was on March 3, 2020, according to sources from the Government and Ministry of Health: https://www.gob.cl/noticias/ministerio-de-salud-confirma-primer-caso-de-coronavirus-en-chile/?utm_source=chatgpt.com. And the gradual reopening of educational centers and time barriers began at the beginning of 2021. https://www.mineduc.cl/mineduc-y-la-unesco-trabajan-para-apoyar-la-reapertura-de-escuelas/?utm_source=chatgpt.com.
